# Recent findings on the global, regional, and national burden of lower extremity peripheral arterial disease in the elderly (1990–2021): an analysis based on the global burden of disease study 2021

**DOI:** 10.3389/fcvm.2026.1688946

**Published:** 2026-02-26

**Authors:** Chenghao Yang, Sheng Fang, Xu Li, Ming Wang, Tianchen Xie, Zhenyu Zhou, Yong Ding, Min Zhou, Zhenyu Shi

**Affiliations:** 1 Department of Vascular Surgery, National Clinical Research Center for Interventional Medicine, Zhongshan Hospital, Institute of Vascular Surgery, Fudan University, Shanghai, China; 2 Department of Vascular Surgery, Shanghai Ninth People’s Hospital, Shanghai JiaoTong University School of Medicine, Shanghai, China

**Keywords:** disability-adjusted life years, global burden of disease, incidence, lower extremity peripheral arterial disease, prevalence

## Abstract

**Introduction:**

Lower extremity peripheral arterial disease (LEPAD) significantly impacts the elderly population, with increasing prevalence globally. This study analyzes the global, regional, and national burden of LEPAD in individuals aged 60 and above from 1990 to 2021, using data from the Global Burden of Disease Study 2021.

**Methods:**

We examined age-standardized prevalence rates (ASPR), incidence rates (ASIR), and disability-adjusted life years (DALYs) across 204 countries. Trends over time were assessed using Estimated Annual Percentage Change (EAPC), and future projections were made through a Bayesian age-period-cohort model.

**Results:**

In 2021, the elderly population experienced 87.5 million LEPAD cases, a 105.5% increase since 1990. ASPR declined slightly (EAPC: −0.52) despite rising absolute cases, reflecting aging populations. High SDI regions had the highest prevalence but also the greatest reductions over time. Regional disparities were notable, with Eastern and Central Europe facing the highest DALYs. Projections to 2040 suggest continued increases in LEPAD burden, with gender disparities persisting.

**Conclusion:**

LEPAD remains a growing global health challenge, driven by aging populations. Targeted interventions are needed, particularly in low-middle SDI regions, to address the escalating burden of LEPAD among the elderly.

## Background

Lower extremity peripheral arterial disease (LEPAD) has emerged as a major health concern disproportionately affecting the elderly population worldwide, with prevalence increasing dramatically in individuals aged 60 years and above. This chronic vascular condition is characterized by the stenosis or occlusion of arteries supplying the lower extremities, resulting in compromised blood flow (1). Beyond its immediate impact on ambulatory function and quality of life, LEPAD serves as a critical indicator of systemic atherosclerosis and elevated cardiovascular risk (2). In the context of global demographic shifts towards an aging population and the increasing prevalence of lifestyle-associated risk factors, a comprehensive understanding of the evolving LEPAD burden is essential for informing public health strategies and optimizing healthcare resource allocation.

The pathophysiology of LEPAD is predominantly attributed to atherosclerosis, with established risk factors including tobacco use, diabetes mellitus, hypertension, dyslipidemia, and advanced age (3, 4). Clinical manifestations range from asymptomatic disease to critical limb ischemia (5). Despite advancements in diagnostic and therapeutic modalities (6, 7), LEPAD remains underdiagnosed and undertreated in many regions, particularly among elderly populations, attributable to limited awareness and inadequate access to specialized vascular care.

The global burden of LEPAD has demonstrated an upward trajectory in recent decades, driven primarily by population aging, with significant heterogeneity across geographical regions and socioeconomic strata (8, 9). The Global Burden of Disease (GBD) Study 2021 provides the most comprehensive assessment of LEPAD, offering crucial insights into its current prevalence, incidence, and associated health burden among elderly populations (10). Understanding these epidemiological patterns is essential for anticipating healthcare needs in aging societies, identifying high-risk elderly populations, and guiding evidence-based public health policies. Given the substantial economic implications of LEPAD in elderly patients (11), our study aims to provide a comprehensive analysis of the global, regional, and national burden of LEPAD specifically in individuals aged 60 years and above using GBD 2021 data, with the objective to inform age-specific interventions and optimize resource allocation for this rapidly growing demographic group.

## Materials and methods

### Data acquisition

The GBD 2021 study offers a comprehensive evaluation of health loss linked to 369 diseases, injuries, and impairments, along with 88 risk factors, across 204 nations and territories, employing the latest epidemiological data and refined standardized methods (10). The GBD database employs sophisticated methods to address missing data and adjust for confounding factors. Details about the study design and methods of GBD studies have been extensively described in existing GBD literature (10). Furthermore, the University of Washington Institutional Review Board waived the requirement for informed consent to access the GBD data (10). This research adhered to the Guidelines for Accurate and Transparent Health Estimates Reporting (GATHER) (12).

### Estimation framework

The GBD study employed sophisticated modeling techniques to estimate the burden of LEPAD. The DisMod-MR 2.1(disease-model-Bayesian meta regression) tool was utilized to calculate incidence and prevalence. This Bayesian geospatial software integrates diverse disease parameters, epidemiological relationships, and geospatial data to generate robust estimates (10). To provide a concise summary of the burden of LEPAD specifically in the elderly population based on age distribution, we classified elderly patients aged 60 years and above into eight groups: 60 to 64 years, 64 to 69 years, 70 to 74 years, 75 to 79 years, 80 to 84 years, 85 to 89 years, 90 to 94 years and 95 plus years. Within the GBD 2021 framework, missing or sparse epidemiological data were addressed using DisMod-MR 2.1, which borrows strength across age, time, and geographical units through Bayesian meta-regression. In this study, we did not apply additional imputation procedures; instead, all estimates relied on the internally modeled results generated by GBD, which incorporate covariate-informed priors and uncertainty propagation to account for data gaps, particularly in regions with limited surveillance data.

All reported estimates are presented with 95% uncertainty intervals (UIs), reflecting uncertainty arising from data availability, model specification, and parameter estimation within the GBD framework. Temporal trends and regional comparisons were interpreted primarily based on point estimates, while uncertainty intervals were used to assess the robustness and overlap of estimates. Conclusions were considered more cautious in regions or age groups with wider uncertainty intervals, indicating greater data sparsity or model uncertainty.

The DisMod-MR 2.1 model assumes consistency in disease epidemiology across regions, which may introduce bias particularly in low- and middle-income countries where underreporting of asymptomatic LEPAD cases in elderly populations is common. The BAPC projection model assumes continuation of observed trends and may not account for emerging risk factors in aging populations (e.g., rising diabetes and obesity prevalence among elderly) or novel therapeutic interventions. These limitations should be considered when interpreting estimates for elderly populations.

### Sociodemographic index

The sociodemographic index (SDI) quantifies a country or region's development level using fertility rate, education level, and per capita income data. Ranging from 0 to 1, a higher SDI indicates greater socioeconomic development (10, 13). The SDI is known to correlate with disease incidence and mortality rates. In this study, we classified countries and regions into five SDI categories (low, low-medium, medium, medium–high, and high) to examine the relationship between LEPAD burden and socioeconomic development.

To assess the trends in age-standardized rates (ASR) of LEPAD incidence, disability adjusted life years (DALYs), and prevalence, the study utilized the Estimated Annual Percentage Change (EAPC). The ASR was computed per 100,000 individuals utilizing the subsequent formula:

$$\mathrm{ASR}=\frac{\sum_{i=1}^{A}\alpha_{i}w_{i}}{\sum_{i=1}^{A}w_{i}}\times 100,000$$

α*_i_*: the age-specific rate in *i*^th^ the age group; w: the number of people in the corresponding *i*^th^ age group among the standard population; *A*: the number of age groups

The EAPC serves as a prevalent metric in epidemiological studies to ascertain temporal evolutions in ASRs of diseases. The calculation of EAPCs was based on a regression model that characterizes the pattern of age-standardized rates during a specified period (14). The equation employed was:

y=α+βx+ε

EAPC=100*(exp(β)−1)

y: the natural logarithm of the ASR; x: the calendar year; α: the intercept term, β: denotes the slope or trend, and ε: the error term.

The linear regression model was used to compute the 95% confidence interval (CI) for the EAPC. An ASR is deemed to have an increasing trend if both the EAPC and the lower bound of its 95% CI are positive. In contrast, if both the EAPC and the upper bound of its 95% CI are negative, the ASR is considered to have a decreasing trend. If neither condition is satisfied, the age-standardized rate is regarded as stable. Spearman correlation was used to assess associations between the SDI and the age-standardized rates of LEPAD.

In this study, we utilized a Bayesian age-period-cohort (BAPC) model incorporating integrated nested Laplace approximations to project future trends in LEPAD burden. Previous research has demonstrated that BAPC offers superior coverage and precision compared to alternative prediction methods (15–18). The computational process was implemented using the R-package BAPC, following established protocols from prior studies (15). All analyses and visualizations were carried out using the World Health Organization's Health Equity Assessment Toolkit and the R statistical computing software (Version 4.3.3).

### Ethics

The institutional review board granted an exemption for this study, as it utilized publicly accessible data that contained no confidential or personally identifiable patient information.

## Results

### Global level

**Figure 1:**
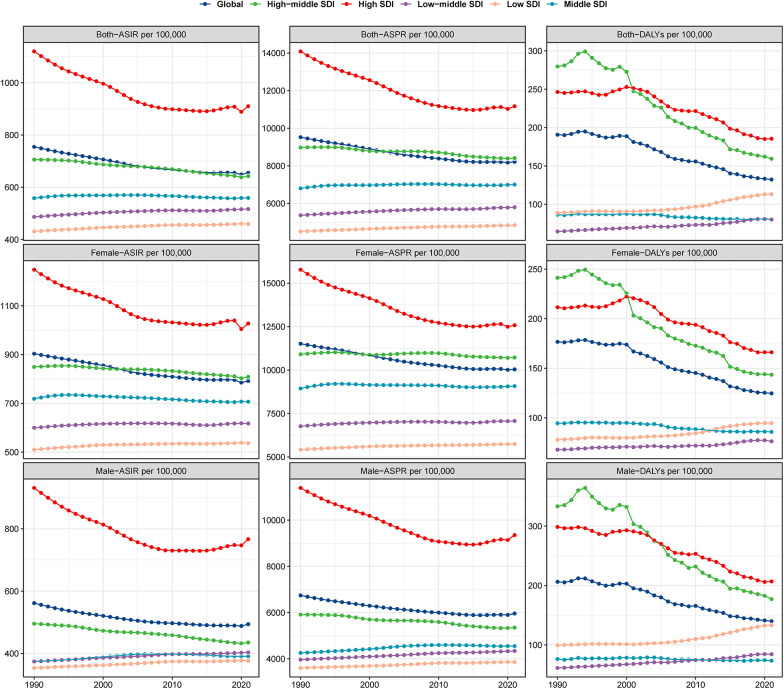
Trends in LEPAD prevalence, incidence and DALYs from 1990 to 2021.

In 2021, the global burden of lower extremity peripheral arterial disease (LEPAD) among individuals aged 60 years and above was substantial, with 87,523,485.6 cases globally, representing a 105.5% increase since 1990. This increase was largely driven by the aging population, though age-standardized prevalence, incidence, and DALYs rates all showed a consistent downward trend (Table 1, Figure 1). Specifically, the age-standardized prevalence rate (ASPR) decreased from 9,525.1 per 100,000 persons in 1990 to 8,220.2 in 2021 (EAPC: −0.52), highlighting a modest but consistent improvement in age-adjusted rates. While this decline is statistically significant, the modest annual percentage change of 0.52% indicates only gradual improvement, despite the increasing absolute number of cases due to population aging. This pattern underscores the growing healthcare demands imposed by elderly LEPAD patients, despite improvements in age-standardized burden. The rise in absolute numbers, especially in regions with rapidly aging populations, indicates an escalating challenge for healthcare systems globally.

**Table 1:** Global and regional trends in lower extremity peripheral arterial disease burden: prevalence, incidence, and disability-adjusted life years (1990–2021).

Location	1990		2021		EAPC_CI
	Number	ASR	Number	ASR	
Prevalence					
Global	42,618,312.9 (34,568,408.9–52,152,591.7)	9,525.1 (7,740.3–11,623.7)	87,523,485.6 (71,462,682.3–106,538,304.1)	8,220.2 (6,717.3–9,995.7)	−0.52 (−0.56–0.48)
High SDI	20,423,876.8 (16,683,602.6–24,867,586.3)	14,087.4 (11,504.2–17,155.5)	32,356,631.6 (26,848,704–38,841,644.7)	11,171.7 (9,262.9–13,431)	−0.83 (−0.93–0.74)
High-middle SDI	10,430,118.5 (8,450,606.5–12,781,382.4)	8,978 (7,284.4–10,970.2)	21,274,030.1 (17,202,896.8–26,043,613.2)	8,411.9 (6,806.4–10,289.7)	−0.24 (−0.26–0.22)
Middle SDI	7,389,048.9 (5,928,991.5–9,142,600.1)	6,807.3 (5,482.1–8,386.9)	22,148,239.1 (17,861,052.8–27,287,112.8)	7,006.9 (5,657.9–8,617.5)	0.04 (0.02–0.06)
Low-middle SDI	3,337,151.5 (2,666,555.6–4,148,532.2)	5,372.8 (4,308.9–6,646.7)	9,218,503.6 (7,384,466.9–11,437,219.3)	5,803.1 (4,659.5–7,178.8)	0.23 (0.21–0.24)
Low SDI	997,208.4 (794,312.1–1,248,912.6)	4,514.9 (3,612.3–5,621.4)	2,454,516.1 (1,961,619.2–3,058,822.4)	4,842.4 (3,880.9–6,010.2)	0.22 (0.21–0.24)
Andean Latin America	94,566 (75,571.5–118,235.3)	4,185.6 (3,347.6–5,224.7)	324,143.9 (258,860.6–402,331.2)	4,578.4 (3,657.6–5,679.8)	0.33 (0.3–0.36)
Australasia	312,596.8 (252,926.2–383,022.9)	10,251 (8,295.7–12,552.5)	536,247.5 (432,678.8–659,090.3)	7,301.2 (5,886.3–8,986.5)	−1.26 (−1.36–1.16)
Caribbean	174,110.3 (138,853.7–216,268)	5,591.1 (4,461.5–6,936.6)	399,743.6 (320,032.2–495,446.9)	5,906.7 (4,729–7,323.4)	0.18 (0.17–0.19)
Central Asia	293,419.1 (234,771.3–364,418.5)	5,564.6 (4,457.1–6,893.2)	531,081.4 (425,604.2–660,024.5)	6,077.4 (4,883.6–7,517.8)	0.38 (0.31–0.45)
Central Europe	1,264,699.1 (1,012,779.1–1,561,321.2)	6,882.2 (5,516.1–8,478.2)	2,096,186.1 (1,690,311.8–2,579,928.2)	6,842.3 (5,514.6–8,425.2)	−0.03 (−0.06–0.01)
Central Latin America	561,837.3 (449,193.1–698,519)	6,239.1 (4,995.9–7,738.4)	1,789,697.7 (1,430,477.2–2,215,304.2)	5,974.4 (4,779.4–7,389.4)	−0.14 (−0.15–0.13)
Central Sub-Saharan Africa	96,432.2 (76,362.6–121,591.2)	4,739 (3,780.1–5,915.4)	236,029.2 (187,622–296,935)	4,878.9 (3,901–6,092.9)	0.08 (0.07–0.09)
East Asia	6,912,972.3 (5,560,460–8,512,163.7)	7,419.3 (5,989.8–9,092.9)	21,232,773.3 (17,173,877.6–26,011,768.3)	7,857.4 (6,361.9–9,610.3)	0.08 (0.03–0.13)
Eastern Europe	2,422,972.4 (1,943,157–2,991,696)	7,075.7 (5,682.9–8,721.6)	3,679,472.8 (2,957,639.5–4,528,234.7)	7,805.4 (6,278.9–9,591.6)	0.46 (0.39–0.52)
Eastern Sub-Saharan Africa	308,105.8 (245,118.5–386,174.6)	4,283.6 (3,424.8–5,337.4)	734,792.4 (584,434.8–919,764.1)	4,528 (3,613.1–5,637.6)	0.18 (0.17–0.19)
High-income Asia Pacific	2,893,767 (2,366,670.1–3,535,685.3)	11,925.5 (9,757.4–14,548.7)	5,275,170.5 (4,281,572.1–6,447,106.4)	7,798.7 (6,317–9,569.3)	−1.57 (−1.66–1.49)
High-income North America	7,812,020.7 (6,322,954.3–9,565,465.5)	16,461 (13,318.2–20,167.8)	13,666,107.8 (11,561,883–16,026,321.5)	15,192.2 (12,855.2–17,819.9)	−0.2 (−0.32–0.08)
North Africa and Middle East	1,854,009.6 (1,485,165–2,314,442.4)	5,378 (4,322.5–6,681.6)	6,141,203.3 (4,927,744.3–7,614,112.4)	6,409.1 (5,154.3–7,914.9)	0.61 (0.58–0.64)
Oceania	18,248.2 (14,639.3–22,779.5)	6,616.6 (5,348.4–8,185.6)	50,623.1 (40,979.7–62,658.9)	7,185.4 (5,840.7–8,839.5)	0.27 (0.24–0.31)
South Asia	5,650,511.6 (4,506,695.1–7,041,205.6)	4,989.8 (3,996.1–6,182.9)	17,797,548.4 (14,241,990.5–22,064,383.2)	5,409.6 (4,338.2–6,688.5)	0.22 (0.19–0.25)
Southeast Asia	2,049,036.7 (1,644,525.3–2,526,908.2)	7,728.6 (6,226.1–9,493.6)	6,084,782.2 (4,883,773.8–7,482,040.7)	8,315.8 (6,695.1–10,190.6)	0.28 (0.23–0.33)
Southern Latin America	576,387.3 (464,858.9–714,017.4)	10,161.8 (8,202.6–12,568.7)	1,044,506.1 (848,392.8–1,287,610)	9,126.8 (7,413–11,255.5)	−0.39 (−0.45–0.34)
Southern Sub-Saharan Africa	214,278.9 (171,355.9–264,747.8)	7,347.4 (5,893.6–9,046.1)	410,084 (328,836.9–508,980.9)	6,645.8 (5,344.6–8,217.5)	−0.42 (−0.47–0.37)
Tropical Latin America	655,070.3 (524,958.9–812,101.6)	6,659.4 (5,350.2–8,226.6)	1,819,333.4 (1,458,940.9–2,254,486.6)	5,804.6 (4,658.7–7,186)	−0.53 (−0.56–0.5)
Western Europe	11,818,505.1 (9,722,850.7–14,361,225)	15,140.5 (12,442.6–18,411.7)	14,742,976.8 (12,038,751.7–18,008,879.8)	11,351.2 (9,257.2–13,908.2)	−1.08 (−1.16–1)
Western Sub-Saharan Africa	387,026.9 (308,924.2–484,170.1)	4,274 (3,420.3–5,324.8)	900,357.9 (719,325–1,122,050.9)	4,708.1 (3,773.8–5,843.5)	0.35 (0.33–0.36)
Incidence					
Global	3,558,759.6 (2,552,524.4–4,802,667.7)	754.7 (538.5–1,020.9)	7,106,685.6 (5,112,437.2–9,558,485.3)	655.4 (470.2–882.7)	−0.49 (−0.53–0.45)
High SDI	1,630,611.9 (1,177,118.1–2,188,782.4)	1,120 (809.7–1,501.9)	2,531,822 (1,858,414.6–3,339,544.6)	910.2 (670.9–1,197.5)	−0.73 (−0.84–0.62)
High-middle SDI	861,944 (614,702.5–1,166,796.4)	706 (501.1–958.1)	1,647,122.4 (1,168,230.3–2,242,212)	642.7 (454.9–875.9)	−0.33 (−0.35–0.32)
Middle SDI	641,191.2 (456,159.7–873,026.1)	558.4 (393.7–763.1)	1,821,801.6 (1,293,382.5–2,484,388.4)	559.2 (395.1–764)	−0.04 (−0.07–0.02)
Low-middle SDI	319,642.3 (228,874.8–432,433.4)	486.6 (345–661.2)	854,443.2 (608,684.2–1,158,594.1)	516.7 (365.3–702.7)	0.17 (0.15–0.19)
Low SDI	101,827.4 (73,227.4–138,117.1)	431 (305.9–587.5)	245,507.3 (176,058.1–333,010.3)	459.5 (326–625.5)	0.2 (0.18–0.22)
Andean Latin America	9,202.8 (6,542–12,490.4)	399.5 (282.9–543)	30,569.4 (21,689.2–41,496.6)	428.5 (303.6–581.8)	0.26 (0.24–0.28)
Australasia	26,754.1 (19,106–35,937)	860.4 (613.7–1,156.2)	44,521 (31,721.4–60,151.5)	620.7 (443.7–837.6)	−1.23 (−1.34–1.13)
Caribbean	16,489.2 (11,679.8–22,520.3)	518 (365.5–708.7)	36,326 (25,632.1–49,265.4)	539.5 (381–731.1)	0.12 (0.11–0.14)
Central Asia	26,630.6 (18,872–36,215.2)	493.6 (348.6–672.6)	48,546.4 (34,480.8–65,692.8)	528.2 (371.6–717.4)	0.28 (0.23–0.33)
Central Europe	110,734.8 (78,412.3–150,685.2)	581.1 (410.9–790.8)	173,907.8 (123,273.7–236,536.6)	570.6 (404.7–775.9)	−0.08 (−0.1–0.06)
Central Latin America	54,111.3 (38,673.9–73,148.2)	579.8 (412.2–785.7)	168,368.2 (120,973.8–228,157.1)	553.3 (396.8–750.2)	−0.14 (−0.15–0.12)
Central Sub-Saharan Africa	10,223.5 (7,363.2–13,835.7)	456.8 (323.3–622)	24,720.1 (17,708.6–33,501.4)	472.6 (334.3–643.3)	0.08 (0.06–0.11)
East Asia	560,249.2 (395,473.6–769,309.3)	563.8 (394.3–776.8)	1,585,331.1 (1,113,575–2,175,988.6)	571.4 (399.9–785.8)	−0.05 (−0.09–0.01)
Eastern Europe	206,396.9 (145,261.6–282,756.5)	582.6 (408.6–799.8)	299,545.5 (211,431.7–408,889.1)	628.3 (441.6–859.8)	0.34 (0.3–0.38)
Eastern Sub-Saharan Africa	32,126.2 (22,999.4–43,524)	416.2 (294–566.5)	74,695.1 (53,146.9–101,651.8)	434.8 (306.4–593.8)	0.14 (0.12–0.15)
High-income Asia Pacific	238,787.6 (170,950.8–320,716.3)	951.9 (679.6–1,280.1)	403,811.7 (284,777.8–549,830.3)	644.5 (458.5–873.9)	−1.44 (−1.52–1.36)
High-income North America	627,617.7 (451,634.6–841,778.5)	1,330.5 (958.6–1,783.2)	1,110,014.3 (839,387.6–1,421,513.7)	1,245.8 (942.1–1,595.6)	−0.11 (−0.27–0.05)
North Africa and Middle East	173,826.9 (123,515.4–236,613)	476.1 (335–651.1)	556,734.7 (396,181.7–754,231.4)	555.5 (392.5–755.3)	0.53 (0.5–0.55)
Oceania	1,675.3 (1,215.8–2,265.6)	554.2 (395.9–753.6)	4,519.3 (3,276.7–6,149.6)	597.1 (427.6–814.1)	0.26 (0.21–0.3)
South Asia	548,242.8 (393,158.9–742,209.7)	455.9 (323.4–620.1)	1,654,867.6 (1,179,312.1–2,245,375.9)	482.6 (341.2–656.9)	0.15 (0.12–0.18)
Southeast Asia	176,182.3 (125,928.6–238,970.7)	629.3 (446–857.1)	500,206.1 (356,697.5–681,391.1)	650.3 (460.2–888.5)	0.14 (0.09–0.18)
Southern Latin America	52,482.4 (37,712.5–70,942.9)	898 (643–1,215.5)	90,224.3 (64,622.2–121,691.3)	795.1 (570.1–1,071.7)	−0.46 (−0.52–0.4)
Southern Sub-Saharan Africa	19,054.9 (13,592.5–25,795.8)	620.8 (439.7–844.1)	37,528.5 (26,654.1–50,944.4)	573.3 (403.7–781.4)	−0.35 (−0.39–0.31)
Tropical Latin America	61,671.3 (44,025.2–83,281.8)	594.9 (421.8–806.3)	165,869.2 (118,032–225,129.6)	522 (370.5–709.2)	−0.5 (−0.53–0.48)
Western Europe	928,540.4 (672,273.9–1,248,002.2)	1,201.6 (873.7–1,610.7)	1,112,844.6 (796,919.8–1,500,205.8)	917.4 (661.3–1,233)	−1.03 (−1.12–0.95)
Western Sub-Saharan Africa	38,794.3 (27,685.4–52,691.6)	407.6 (287.8–555.7)	89,335.9 (63,875.6–120,672.4)	444.2 (314.4–602.8)	0.31 (0.29–0.32)
Disability-adjusted life years					
Global	797,939 (640,654.4–1,050,371.2)	190.7 (153.8–247)	1,380,105 (1,071,954.7–1,859,460.5)	132.3 (102.9–177.2)	−1.4 (−1.51–1.3)
High SDI	350,256.6 (287,926.2–449,443.4)	246.4 (202.3–315.3)	560,503.1 (452,424.5–710,642.3)	185.5 (150.2–236.7)	−1.04 (−1.19–0.89)
High-middle SDI	303,108.8 (254,909.9–371,096.9)	279.6 (235.1–339)	396,569 (321,395.8–514,985.5)	159.4 (129.2–206.1)	−2.23 (−2.39–2.08)
Middle SDI	86,221 (57,214.2–137,094.9)	86.5 (58.7–133.5)	244,513.4 (167,729.6–376,741.8)	80.4 (55.5–122.2)	−0.33 (−0.38–0.28)
Low-middle SDI	38,193.7 (22,523.4–62,728.1)	64.7 (38.5–104.3)	122,032.2 (82,426.4–183,936.5)	80.2 (54.4–119.3)	0.69 (0.65–0.74)
Low SDI	18,774.8 (9,346.4–32,413.9)	88.9 (44.1–154.8)	54,067.6 (29,888.1–90,737.4)	113.2 (62.5–191.4)	0.8 (0.68–0.92)
Andean Latin America	789.6 (425.6–1,411)	35.5 (19.2–63)	2,886.7 (1,792.8–4,788.1)	41 (25.5–67.9)	0.59 (0.45–0.73)
Australasia	11,020.7 (9,441.4–12,861.9)	383.2 (327.2–445.8)	14,328.1 (11,419.1–17,505)	181.8 (145.1–223.4)	−2.62 (−2.82–2.42)
Caribbean	6,124.8 (5,252.4–7,320.7)	208.2 (178.6–247.4)	16,550.5 (13,967.2–19,684)	241.3 (203.9–286.9)	0.54 (0.44–0.64)
Central Asia	3,109.9 (2,029.9–4,872.9)	60 (39–94)	7,267.7 (5,320.2–10,383.6)	87.2 (64.2–123.3)	1.42 (1.28–1.57)
Central Europe	58,713 (52,099.3–67,173.3)	333 (294.6–380.4)	100,926.9 (86,684.2–117,148.2)	329.8 (283.1–383)	−0.47 (−0.71–0.23)
C entral Latin America	9,875.2 (7,707.8–13,424.6)	116.9 (92–156.6)	23,151.8 (16,939.9–33,663.1)	78.2 (57.4–113.3)	−1.55 (−1.78–1.32)
Central Sub-Saharan Africa	3,502.8 (1,729.6–6,377.7)	176.4 (87.5–321.9)	11,314.6 (6,346.9–19,755.4)	239.9 (134.2–422.8)	1.05 (0.79–1.3)
East Asia	57,010.5 (29,682.9–105,527.7)	64.7 (34–117.4)	159,480.4 (87,895.8–285,259.5)	60.5 (33.7–106.8)	−0.34 (−0.4–0.28)
Eastern Europe	170,202.4 (147,151.9–197,750.5)	514.9 (442.6–597.3)	172,098.6 (148,693.9–200,562.6)	363.8 (314.1–424.3)	−1.66 (−1.93–1.39)
Eastern Sub-Saharan Africa	8,207.7 (4,198.8–14,783.4)	120.2 (61.3–217.1)	24,718.4 (14,744.9–44,269.5)	160.9 (95.5–286.6)	0.97 (0.87–1.07)
High-income Asia Pacific	22,660.8 (14,680.7–36,551)	96.9 (63.6–154.2)	52,222.2 (35,855.2–76,278)	69 (46.9–104.1)	−1.11 (−1.26–0.97)
High-income North America	121,068.1 (99,369.1–154,130.3)	256.2 (210.2–325.9)	220,340.2 (179,838.4–276,850.5)	241.4 (197.5–303.5)	−0.67 (−0.9–0.44)
North Africa and Middle East	18,660.9 (10,932–31,158.3)	55.1 (32.3–91.3)	60,914.3 (39,856.3–95,698.6)	66.3 (43.6–102.8)	0.86 (0.72–1.01)
Oceania	151.6 (75.8–287.1)	57.6 (29–106.7)	420.7 (216.2–784.8)	62 (32–113.8)	0.2 (0.09–0.3)
South Asia	54,093.4 (29,226.6–95,433.8)	49.4 (26.7–86.1)	194,301.1 (118,805.5–313,198.2)	61.6 (37.9–98.3)	0.76 (0.71–0.8)
Southeast Asia	16,916.4 (8,848.2–31,232.7)	65.2 (33.9–119.6)	49,232.8 (27,091.2–88,246.7)	69 (37.8–122.8)	0.21 (0.16–0.25)
Southern Latin America	6,504.9 (4,627–9,789.2)	117.5 (84.2–175.1)	11,325.2 (8,167.2–16,670.2)	98.3 (70.8–145)	−0.39 (−0.64–0.14)
Southern Sub-Saharan Africa	5,237 (3,782.3–7,076)	182.2 (130.7–245.8)	15,903.3 (13,243.7–19,170.5)	258.3 (214.5–311.8)	1.34 (1.12–1.56)
Tropical Latin America	19,067.2 (16,319.1–23,223.5)	203.5 (173.5–246.3)	55,909.4 (47,354.5–67,149.7)	179.2 (151.5–215.1)	−0.63 (−0.82–0.44)
Western Europe	230,297 (189,604.1–293,318)	299.1 (245.6–380.8)	282,404.5 (224,945.1–356,361.4)	200.6 (160.1–257)	−1.16 (−1.37–0.94)
Western Sub-Saharan Africa	11,102.3 (6,042.9–19,998.8)	132.7 (72–240.5)	32,015.1 (18,686.2–58,588.3)	182.1 (104.6–334.1)	1.02 (0.99–1.05)

Table 1 Global and regional trends in LEPAD burden: prevalence, incidence, mortality, and DALYs (1990–2021).

Additional File S1 (.doc): National trends in Lower extremity peripheral arterial disease burden: prevalence, incidence, and disability-adjusted life years (1990–2021).

### Regional level

**Figure 2:**
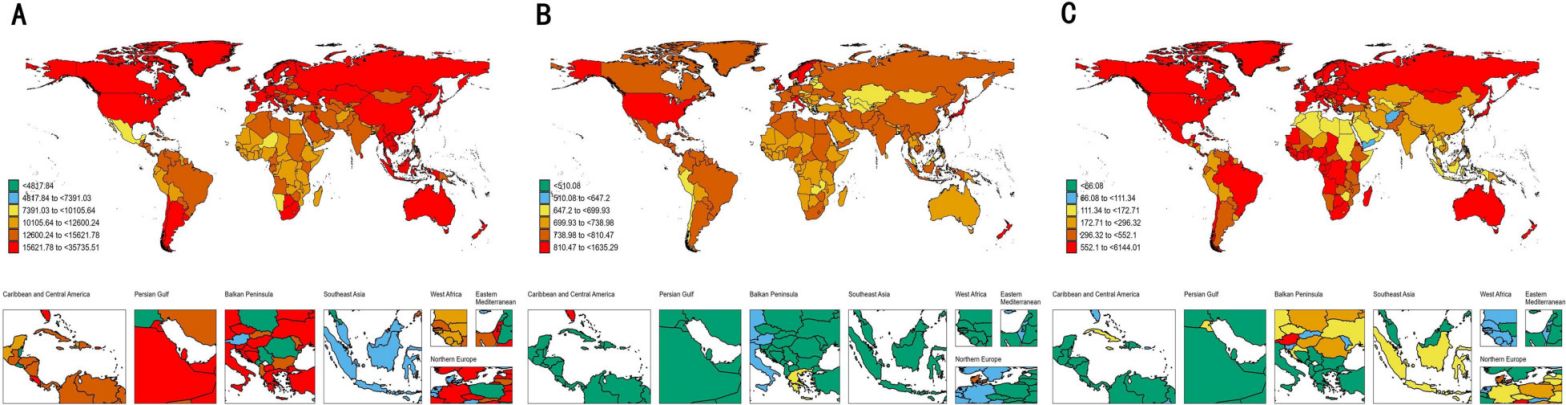
The global disease burden of LEPAD for both sexes in 204 countries and territories. **(A)** Prevalence rate. **(B)** Incidence rate. **(C)** DALYs rate.

**Figure 3:**
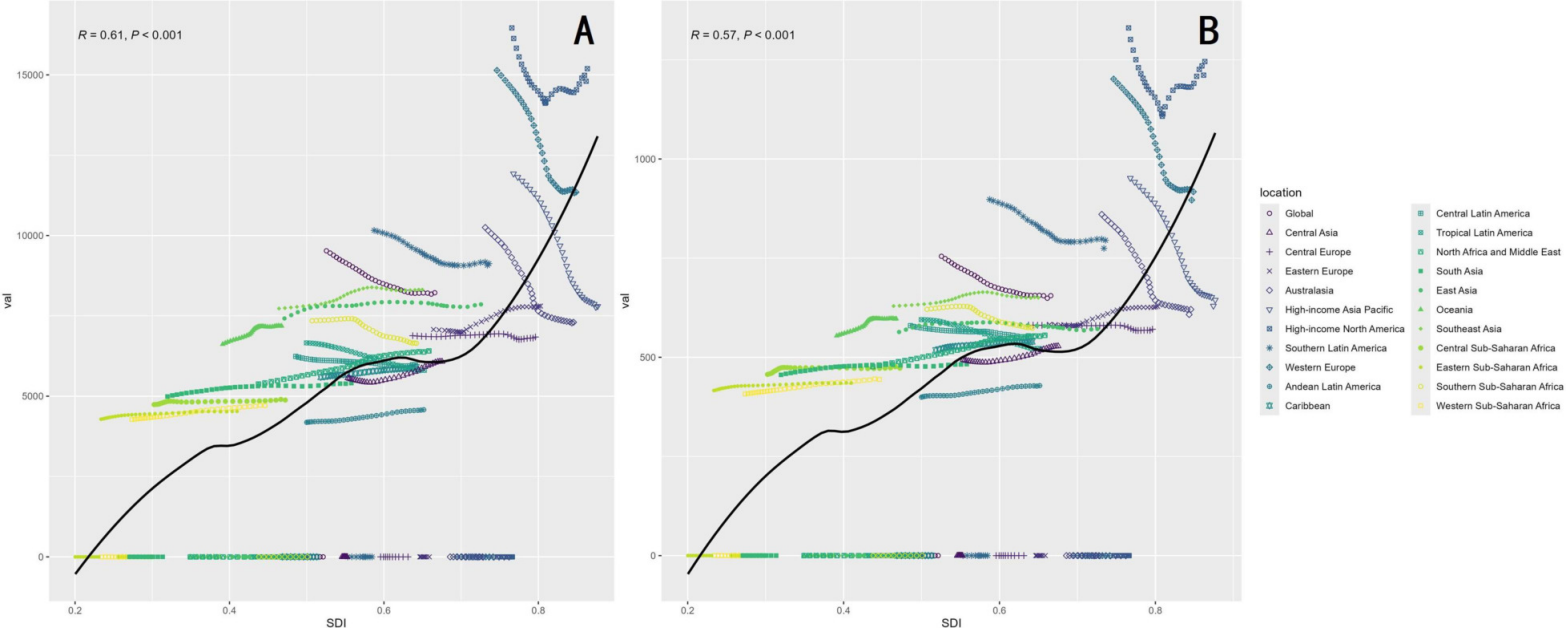
Association between the age-standardized prevalence rate and incidence rate of lower extremity artery disease and the socio-demographic index in 2021. **(A)** Prevalence vs SDI. **(B)** Incidence vs SDI.

Marked regional disparities in LEPAD burden were observed across sociodemographic index (SDI) levels (Table 1, Figure 2). High SDI regions exhibited the highest age-standardized prevalence and incidence rates (Figure 3), with an ASPR of 11,171.7 per 100,000 persons in 2021, but also achieved the most pronounced declines over time, with an EAPC of −0.83. These regions, which include North America and Western Europe, have benefited from better healthcare infrastructure, enhanced diagnostics, and public health policies aimed at reducing risk factors. In contrast, low-middle SDI regions saw sustained increases in age-standardized prevalence (EAPC: 0.23), with ASPR rising from 4,842.4 per 100,000 in 1990 to 5,289.8 in 2021 (Figure 2A). This rise can be attributed to several factors, including increasing life expectancy, urbanization, and greater exposure to cardiovascular risk factors like obesity and smoking.

Age-standardized DALYs rates also varied considerably across regions (Table 1, Figure 2C). Eastern and Central Europe experienced the highest DALY burden, with an age-standardized DALY rate of 363.8 per 100,000 persons in Eastern Europe in 2021, while Western Europe reported a rate of 185.5 per 100,000 persons. The higher DALYs in Eastern Europe suggest that LEPAD in these regions leads to more severe disability and less effective secondary prevention compared to wealthier regions. Conversely, regions in Latin America and Southeast Asia exhibited comparatively lower DALYs rates, with rates of 37.8 and 25.5 per 100,000, respectively (Figure 2C). These differences reflect the varying stages of epidemiological transition and access to healthcare across regions.

At the regional scale, North America and Western Europe exhibited the highest prevalence and incidence rates in 2021 (Figures 2A,B). However, Western Europe experienced more pronounced reductions in both prevalence and incidence over time compared with North America (Table 1), indicating greater success in long-term cardiovascular risk reduction and chronic disease management. These regional differences highlight the role of healthcare system organization, public health interventions, and lifestyle factors in shaping LEPAD outcomes.

### National level

At the national level, the United States had the highest age-standardized prevalence rate of LEPAD among elderly individuals, with an ASPR of 15,577.5 per 100,000 persons in 2021. Denmark and Luxembourg followed closely with rates of 14,188.1 and 12,660.5 per 100,000 persons, respectively (Supplementary Table S1, Figure 2A). These countries reflect a combination of high diagnostic capacity, aging populations, and lifestyle risk factors. Notably, the highest DALYs rates were concentrated in several Eastern and Central European countries, including Hungary (763.1 per 100,000 persons) and the Russian Federation (600.8 per 100,000 persons), highlighting a greater disability burden despite relatively lower prevalence rates (Figure 2C). Countries in parts of Africa and the Middle East exhibited the lowest DALYs rates, though these findings are likely influenced by underreporting and limited data availability.

### Age and sex patterns

**Figure 4:**
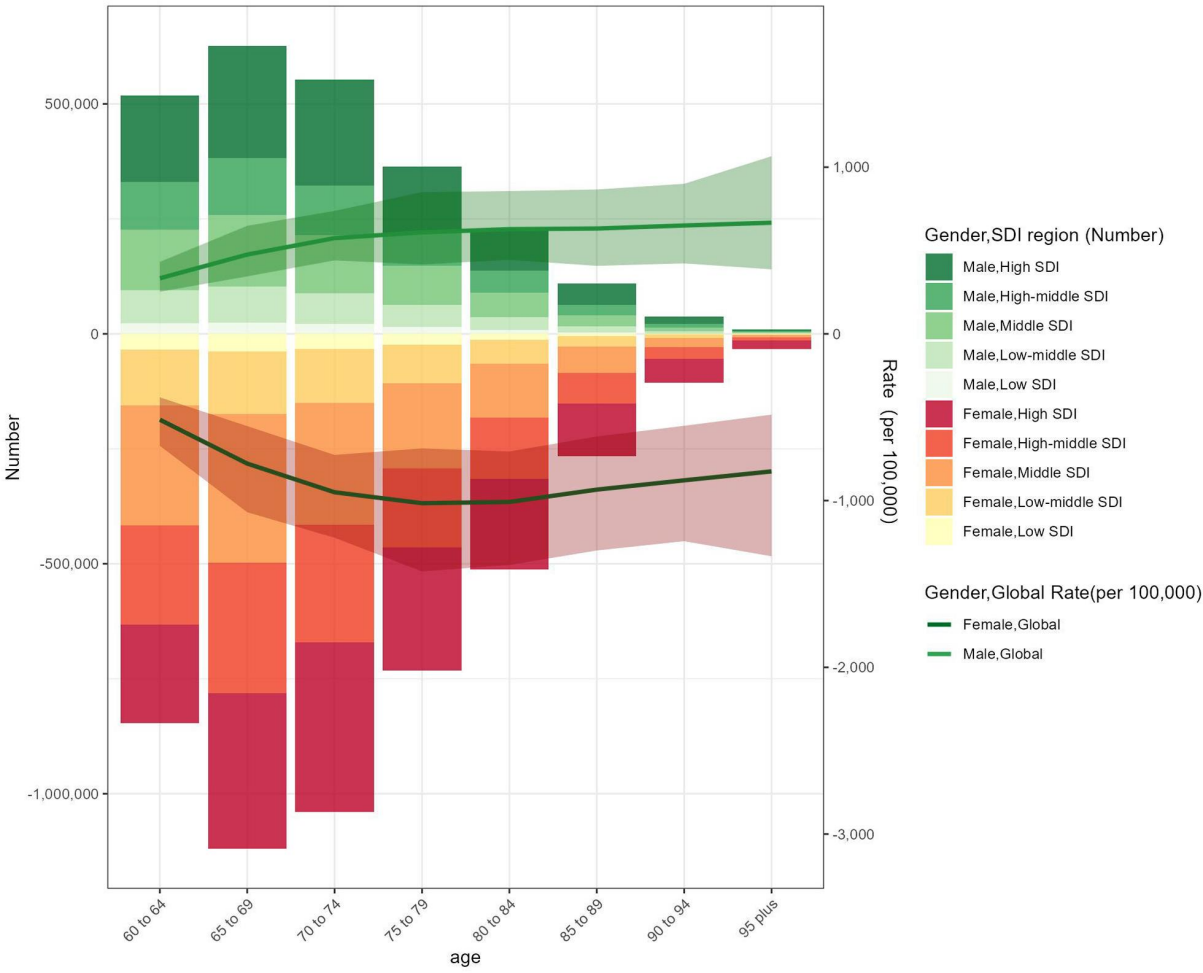
The age-specific numbers and ASIRs of LEPAD by SDI regions in 2021.

**Figure 5:**
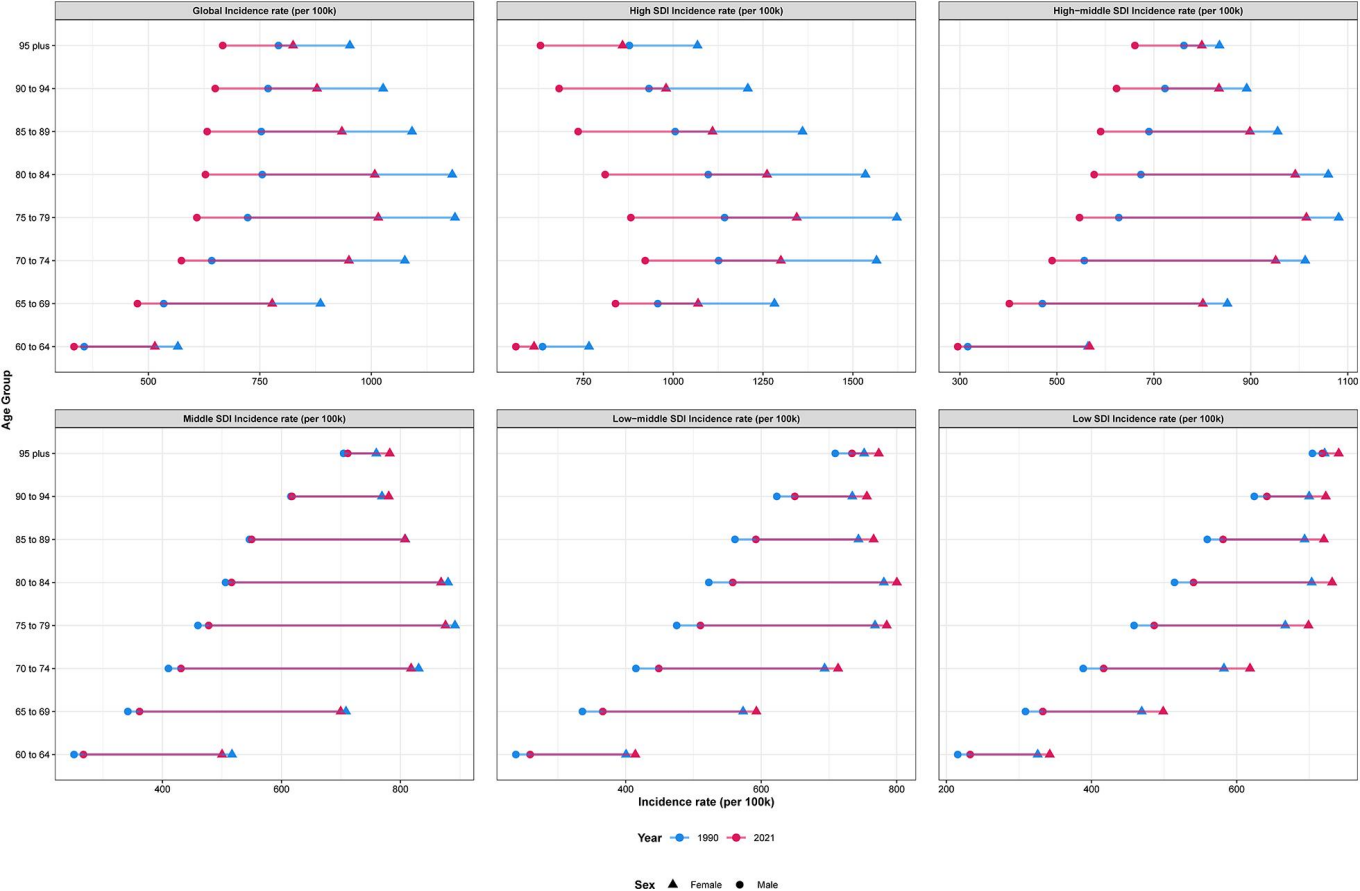
ASIRs of LEPAD by sex, age group, and SDI, 1990 and 2021.

**Figure 6:**
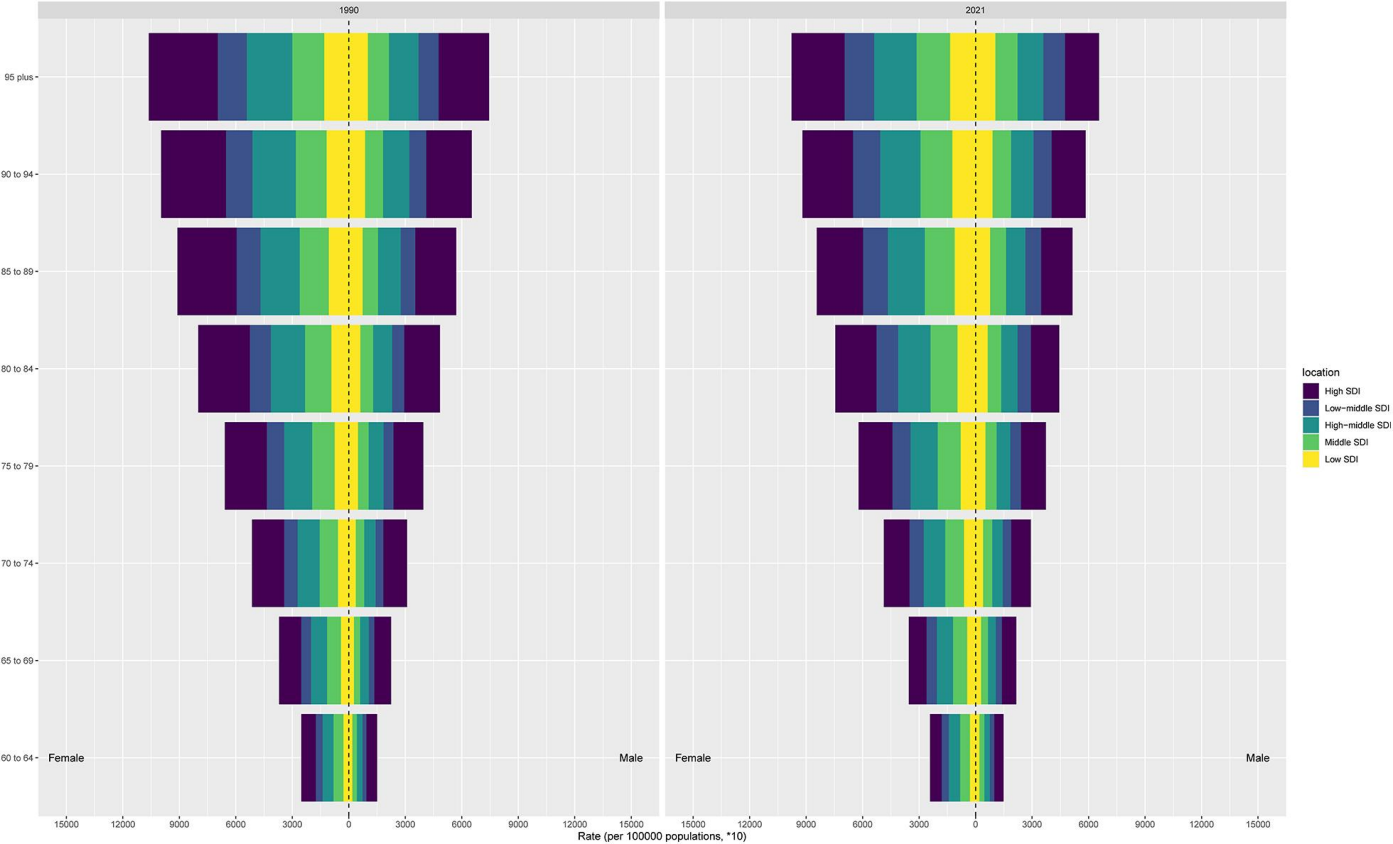
ASPRs of LEPAD by sex, age group, and SDI, 1990 and 2021.

LEPAD incidence and prevalence both increased sharply with advancing age, particularly among those aged 80 years and above (Figure 4). This age gradient was consistent across all regions, with the highest incidence observed in the oldest-old population (Figure 5). Males consistently exhibited higher incidence, prevalence, and DALYs rates than females, with males showing an ASPR of 9,852.6 per 100,000 in 2021 compared to 6,132.3 for females in high SDI regions (Figures 5, 6). This sex disparity is likely due to higher exposure to risk factors such as smoking and hypertension in males, as well as physiological differences that may influence disease progression. Despite the overall increase in burden between 1990 and 2021, these age- and sex-specific patterns remained relatively stable, suggesting that the risk of LEPAD is disproportionately affecting older males in particular.

### Future forecasts of global burden of LEPAD

**Figure 7:**
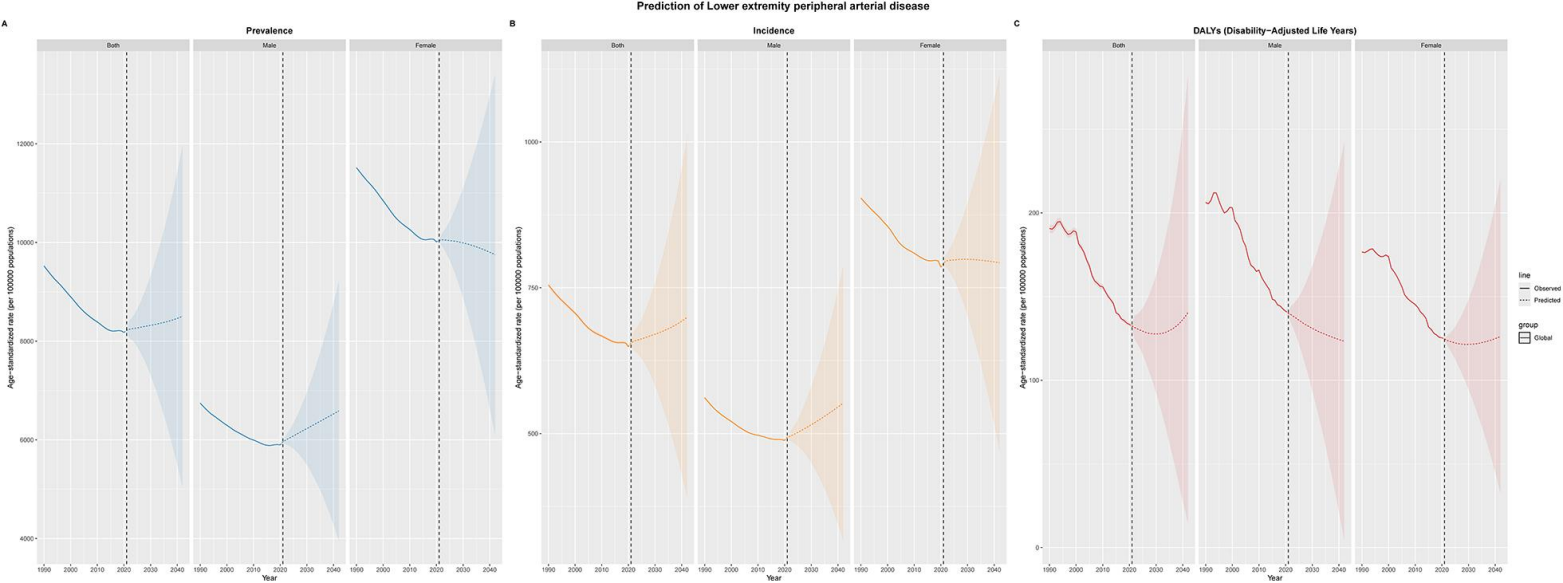
Future forecasts of global burden of LEPAD. **(A)** Global distribution of prevalence. **(B)** Global distribution of incidence. **(C)** Global distribution of DALYs.

Projections to 2040 suggest that the global burden of LEPAD among the elderly population will continue to rise in absolute terms, driven primarily by ongoing population aging (Figure 7). The age-standardized prevalence rate is expected to increase slightly from 8,220.2 per 100,000 in 2021 to about 8,462 per 100,000 by 2040. Similarly, the incidence rate is projected to increase from 655 per 100,000 in 2021 to 693 per 100,000 by 2040, reflecting a steady rise in LEPAD cases as the elderly population expands. These projections suggest that, despite improvements in age-adjusted risk factors, the expanding elderly population will continue to drive an increase in the total burden of LEPAD globally, placing significant pressure on healthcare systems worldwide.

## Discussion

Our comprehensive analysis of LEPAD in the elderly population aged 60 years and above reveals a paradoxical epidemiological pattern: dramatically increasing absolute case numbers driven by population aging, despite concurrent declining age-standardized rates. The global prevalence increased by 105.5% from 1990 to 2021, yet the age-standardized prevalence rate declined by 0.52% annually. This reflects successful cardiovascular risk management over three decades, even as demographic shifts create expanding healthcare burdens. The pattern underscores a critical challenge: improving disease prevention does not necessarily reduce healthcare demand when populations are aging rapidly.

The declining age-standardized rates merit careful examination. Substantial reductions in cardiovascular risk factors documented globally likely contribute significantly. The NCD Risk Factor Collaboration demonstrated that age-standardized prevalence of daily smoking decreased by 28.4% in males and 34.4% in females globally between 1990 and 2019 (19). Similarly, improvements in hypertension control and diabetes management have been documented across many regions (20, 21). These reductions translate directly into lower LEPAD incidence at the population level. However, elevated rates in certain regions suggest that risk factor control remains inadequate where healthcare access is limited or lifestyle transitions occur rapidly.

The striking regional variations reflect fundamentally different stages of epidemiological transition. High SDI regions demonstrate the highest age-standardized prevalence rates yet show the most pronounced declines (EAPC −0.83). This emerges from converging factors: sophisticated diagnostic infrastructure identifying LEPAD more effectively, improved survival following cardiovascular events allowing longer life with atherosclerotic disease, and the oldest populations globally with larger proportions aged 80+ years. Hirsch and colleagues demonstrated that systematic screening identified LEPAD in 29% of individuals over 70 years with cardiovascular risk factors (22).

Conversely, low-middle SDI regions experienced the greatest increases in age-standardized rates (EAPC 0.23), signaling concerning epidemiological transition. These regions face dual challenges: rapidly aging populations combined with increasing cardiovascular risk factor exposure through urbanization. This transition occurs with unprecedented speed, outpacing healthcare infrastructure development needed for chronic disease management (23). Rising burden represents convergence of demographic aging, increased risk factor exposure particularly from tobacco and unhealthy diets, and insufficient healthcare capacity.

The substantial regional variations in LEPAD burden among elderly populations reflect complex interactions between healthcare system capacity, socioeconomic development, and population aging dynamics. In high SDI regions, elevated prevalence despite advanced healthcare reflects improved diagnostic capabilities and longer survival, enabling more elderly individuals to live with LEPAD. A study by Hirsch et al. demonstrated that systematic screening identified LEPAD in 29% of elderly patients over 70 years with cardiovascular risk factors in high-income settings (22). Conversely, the rising burden in low-middle SDI regions stems from rapid epidemiological transition, with elderly populations facing dual challenges of emerging cardiovascular risk factors and limited access to specialized vascular care (24). The disproportionately high DALYs in Eastern and Central Europe among elderly reflect persistent socioeconomic challenges post-Soviet transition, including reduced healthcare funding and delayed adoption of modern preventive strategies specific to aging populations (25). Western Europe's success in reducing elderly LEPAD burden compared to North America can be attributed to universal healthcare coverage, stronger primary care systems enabling earlier detection in older adults, and comprehensive cardiovascular risk reduction programs targeting the elderly.

The contrast between North America and Western Europe provides important insights into healthcare system influences on population health. Western Europe achieved substantially greater LEPAD burden reductions (EAPC −1.08 vs. −0.20 in North America). This difference cannot be attributed to diagnostic practices alone. Western European healthcare systems typically provide universal coverage with strong primary care gatekeeping, facilitating continuous cardiovascular risk management (26). The Netherlands and Norway achieve near-complete population coverage with integrated primary care emphasizing preventive services. In contrast, the United States has historically had fragmented coverage and weaker primary care infrastructure (27). Additionally, Western European countries implemented comprehensive population-level interventions including smoking bans, taxation on unhealthy products, and urban planning promoting physical activity (28).

The disproportionately high DALYs burden in Eastern and Central Europe reflects persistent challenges from post-Soviet healthcare transitions and entrenched cardiovascular risk factors. These regions experienced substantial healthcare system disruptions following Soviet Union dissolution, with reduced public health funding and delayed preventive strategy adoption (25). Limited healthcare resources combined with high risk factor exposure creates conditions where LEPAD develops earlier and progresses more rapidly. Socioeconomic instability contributed to delayed care-seeking and reduced medication adherence (29).

The pronounced age gradient, with incidence rising sharply after 80 years, reflects atherosclerosis's cumulative nature and age-related vascular changes. Atherosclerosis develops over decades through repeated vascular injury and inflammatory responses (30). The oldest-old have survived long enough for these processes to manifest clinically and experience age-related changes including increased arterial stiffness and endothelial dysfunction (31). LEPAD burden concentration in oldest age groups has important healthcare planning implications, as these individuals typically have multiple comorbidities and frailty complicating treatment decisions. As populations continue aging globally, healthcare systems must develop geriatric-focused approaches accounting for functional status and treatment goals beyond purely anatomic revascularization.

The persistent gender disparity, with males showing consistently higher rates across all age groups and regions, likely reflects biological and behavioral factors. Males have higher lifetime tobacco exposure, with smoking rates substantially exceeding females in most regions historically (19). Additionally, males demonstrate higher prevalence of poorly controlled hypertension and differential healthcare-seeking behaviors. Physiological differences may also contribute, as estrogen appears to confer vascular protection in premenopausal women (32). The gender gap narrows in oldest age groups, suggesting female survival to advanced ages selects for individuals with vascular disease burden comparable to males.

Our projections to 2040 indicate continued increases in absolute LEPAD burden, with age-standardized rates remaining relatively stable. This reflects demographic aging certainty, as individuals born during mid-20th century high-fertility periods reach advanced ages over the next two decades. Even with continued risk factor management improvements, elderly population expansion will drive increasing healthcare demands. Projected widening gender disparities suggests current prevention efforts may be more effective in females or that male-specific risk factors prove more resistant to intervention.

Compared to previous GBD-based analyses, our study provides several advances. While GBD 2019 PAD analysis provided comprehensive global estimates, our specific focus on elderly populations aged 60+ represents the demographic accounting for vast majority of LEPAD burden (18). By restricting analysis to elderly populations, we capture the true magnitude of age-related disease burden and provide more targeted insights for geriatric healthcare planning. Our analysis extends to 2021, capturing recent trends including potential COVID-19 pandemic impacts. More importantly, our granular regional analysis reveals patterns obscured in global summaries, particularly diverging trajectories between high and low-middle SDI regions, with important implications for resource allocation. Our projections to 2040 specifically for elderly populations provide actionable forecasts for healthcare planning.

Several limitations warrant consideration. GBD modeling relies on data quality varying substantially across countries, with low-income regions having sparser data. Asymptomatic LEPAD, comprising substantial disease burden particularly in elderly populations, is likely underreported in regions with limited screening access (33). Standardized case definitions may not capture regional variations in diagnostic criteria. Age groupings may obscure important heterogeneity within strata, particularly among oldest-old where functional status varies tremendously. Projections assume continuation of current trends and do not account for potential disruptions including novel therapeutics or unforeseen demographic shifts.

Despite limitations, findings have clear policy implications. Healthcare systems must prepare for growing elderly LEPAD patient volumes, requiring diagnostic and therapeutic capacity expansion. Primary care strengthening emerges as critical, as early detection and risk factor management prevent progression. Targeted screening for high-risk elderly individuals, particularly males aged 60–80 with cardiovascular risk factors, could identify disease earlier when interventions are most effective. Low-middle SDI regions require particular attention, facing rapidly increasing burden with limited resources, suggesting need for international cooperation. Western Europe's success demonstrates that population-level prevention is achievable through comprehensive approaches, providing a model for other regions. The burden concentration in oldest-old highlights need for geriatric-focused approaches prioritizing functional outcomes and quality of life.

Future research should address critical knowledge gaps. Longitudinal cohort studies in elderly populations are needed to understand LEPAD natural history in advanced age. Comparative effectiveness research examining different management strategies in elderly patients could inform treatment decisions where randomized trial evidence is limited. Implementation research exploring effective LEPAD prevention and care delivery in low-resource settings would provide actionable strategies. Studies examining multimorbidity and frailty impacts on LEPAD outcomes could refine risk stratification. Health economic analyses quantifying cost-effectiveness of different strategies specifically in elderly populations would inform resource allocation as healthcare systems face competing demands from aging populations.

## Conclusion

Our analysis reveals LEPAD in the elderly population (aged ≥60 years) as a complex global health challenge fundamentally driven by population aging, with absolute disease burden escalating despite improvements in age-standardized rates. Regional variations reflect the interplay of socioeconomic development, healthcare system capacity, and aging demographics, with high SDI regions managing LEPAD in increasingly older populations while low-middle SDI regions face dual challenges of rapid aging and limited geriatric vascular care. The projected increase in elderly LEPAD cases by 2040, particularly among the oldest-old (≥80 years), underscores urgent needs for age-specific prevention strategies and healthcare system preparedness for the growing elderly population with LEPAD across diverse socioeconomic contexts.

## Data Availability

The datasets presented in this study can be found in online repositories. The names of the repository/repositories and accession number(s) can be found in the article/Supplementary Material.
